# Editorial: Systemic lupus erythematosus - predisposition factors, pathogenesis, diagnosis, treatment and disease models

**DOI:** 10.3389/fimmu.2022.1118180

**Published:** 2022-12-16

**Authors:** Kutty Selva Nandakumar, Kerstin Nündel

**Affiliations:** ^1^ Department of Environmental and Biosciences, School of Business, Innovation and Sustainability, Halmstad University, Halmstad, Sweden; ^2^ Department of Medicine, University of Massachusetts Chan Medical School, Worcester, MA, United States

**Keywords:** inflammation, B cells, autoantibodies, immune complex, systemic lupus erythemaotsus

Systemic Lupus Erythematosus (SLE) is a systemic autoimmune disease affecting multiple organs characterized by a complex interplay of immune cells, factors and pathways resulting in various clinical manifestations. SLE pathogenesis includes a compromised clearance of nucleic acids, increased type I interferon (IFN) response, dysregulated B cell tolerance causing an increased autoantibody synthesis, immune complex formation and deposition, resulting in multi-organ damage. Several susceptibility loci including HLA and non-HLA genes contribute to lupus development and novel loci are being reported continuously from different regions (1). Genetic and environmental factors as well as stochastic events are involved in the disease initiation and perpetuation. In this collection, using genome-wide association study in Han Chinese population, Song et al., identified a most significant associating SNP rs12606116 (18p11.32) and involvement of TGF-β in lupus nephritis, one of the common and serious complications of SLE. In the genetically susceptible individuals, some viruses (Epstein-Barr virus (EBV), parvovirus B19, and human endogenous retroviruses) may play the role of environmental triggers contributing to lupus pathogenesis. Laurynenka et al. have identified high prevalence of anti-EBNA1 heteroantibodies in SLE patients, which reconfirmed the association between EBV infection and SLE development.

Complexity in the clinical features of SLE indicates presence of several subsets of SLE, with an underlying unique combination of disease pathways, genes and environmental factors. However, treatment is broadly based on NSAIDs, corticosteroids, antimalarial drugs, immunosuppressants and biologics. Surprisingly, only two new therapies were approved over the last decade. Therefore, it is essential to understand the basic mechanisms of disease development to focus on developing specific treatments directed toward different subsets of SLE. So, we initiated a collection of articles to summarize research findings, which could form a basis for further exploring genetic and environmental factors of SLE, new diagnostic procedures, pathogeneic mechanisms and treatment modalities. Twenty original and review articles together were published in this collection highlighting a wide range of topics in lupus research.

The pathogenesis of SLE is closely related to a hyperactivation of different immune cells, like T-, B- cells and monocytes with abnormally methylated differentially expressed genes (2). Presence of autoreactive T cells in both human and murine SLE, indicates an imbalance between pathogenic and regulatory T cells. Although most studies related to T cell balance focused on CD4^+^FoxP3^+^T cells (CD4^+^Tregs), recently a non-redundant role of CD8^+^ Tregs (CD44^+^CD122^+^Ly49^+^) was highlighted in regulating lupus-like disease facilitated by the transcription factor Helios, a zinc finger protein and member of the Ikaros family. For self-tolerance and prevention of SLE-like disease, inhibition of T_FH_ cells by CD8^+^ Tregs was found to be essential (3). París-Muñoz et al. have found down regulation of Helios expression in CD8^+^ Tregs during lupus progression in mice.

B cells, on the other hand, have a crucial contribution to the disease development by not only secreting antibodies targeted to nuclear antigens but also in antibody-independent mechanisms including antigen presentation, regulation of other immune cell functions and synthesis of various cytokines. In general, B cells are subclassified into different subsets: B1, B2, marginal zone (MZ) B cells and B-regs. B2 cells (follicular B cells) produce specific, high-affinity antibodies against foreign antigens by cooperating with T cells in the germinal centers (GCs) and involved in immunological memory. Whereas MZ B cells secrete IgM antibodies and the self-renewing B1 cells produce germline encoded “natural antibodies” mainly of low affinity, multi-reactive IgM isotype, as part of the innate immunity, which are important in anti-microbial immunity and house-keeping roles such as clearing apoptotic, and necrotic cells. Autoantigens released because of the defects in cell death pathways like apoptosis and NETosis could be presented by follicular dendritic cells to autoreactive B cells in GCs resulting in loss of self-tolerance causing production of autoantibodies, immune complexes, and pro-inflammatory cytokines leading to inflammation and tissue damage in SLE (4). In their review, Zhou et al. have focused on the contribution of B1 cells to SLE pathogenesis.

In the immune cells of SLE patients and lupus animals, an abnormal expression of tyrosine kinases was observed. Based on structure, tyrosine kinases are classified either as receptor or as non-receptor kinases. The expression level of Src family of non-receptor protein tyrosine kinases, forms an important determinant of immune tolerance. Among them, Lyn has a regulatory role in the signaling pathways within B cells as well as other hematopoietic cells. Lyn initiated negative signaling is crucial in B cell activation and Lyn deficient mice developed SLE-like disease with plasma cell hyperplasia (5). CD11b, the α-chain of integrin receptor CD11b/CD18 (αMβ2, Mac-1), is highly expressed on the surface of innate immune cells. Genetic variants in the human *ITGAM* gene encoding this CD11b protein are strongly associated with SLE (6). Gottschalk et al. have reported deficiency in CD11b accelerated nephritis in Lyn deficient mice suggesting a protective role of CD11b in lupus inflammation.

Bruton’s tyrosine kinase (Btk) belongs to the Tec family of tyrosine kinases, which modulates development, selection, activation and survival of B cells (7), apart from regulating signaling in myeloid cells. Transgenic mice overexpressing Btk in B cells spontaneously develop SLE-like autoimmune pathology (8). Du et al. have studied the therapeutic effect of a Btk inhibitor (BTKB66) using four models of end-organ inflammation. Interestingly in another study, Schall et al. have convincingly demonstrated a synthetic peptide (P140) binding to HSPA8/HSC70 chaperone protein has the capacity to clear most of the hyper-activated T and B cells in lupus mice, and thus provide a new and promising therapeutic candidate for clinical application.

Autoantibodies secreted by long-lived plasma cells is one of the distinct features of SLE. Also, an association between SLE and type I interferon regulated genes is well known (9). In this context, Akita et al. characterized the plasmablasts (CD38^+^CD43^+^ B cells) from SLE patients and found an increased expression of type I IFN-stimulated genes, in addition to cell cycle-related genes associated with the transcriptional factor, FOXM1, a regulator for cell proliferation and survival. The authors suggested FOXM1 inhibitor may have more anti-proliferative and cytotoxic effects on lupus associated plasmablasts. Autoantibodies either individually or as a part of immune complexes activate the immune system by cross-linking the Fc part of IgG (FcγR) expressed on the immune cells and initiating the activation of complement cascade (10). In the distal region on chromosome 1, genes of four activating FcγRs (CD64, CD16A, CD16B, and CD32A) are clustered and associated with susceptibility to autoimmune diseases like lupus (11) and arthritis (12) in humans. Similarly, in mice the FcγR genes (II, -III, and -IV) associated with lupus-like disease are present in a clustered region orthologous with SLE associated genomic intervals in humans, whereas the activating receptor FcγRI is located on another chromosome because of a translocation during evolution. CD64 (FcγRI) is the only known high-affinity FcγR for IgG with a restricted isotype specificity. Jiang et al. have observed an improvement in lupus-serum induced skin inflammation in CD64 deficient mice suggesting CD64 might be used as a possible biomarker for monitoring SLE development. In both mice and humans, the activating FcγRs are counterbalanced by one inhibitory receptor FcγRIIb (CD32B) with an immunoreceptor tyrosine-based inhibition motif (ITIM) within its cytoplasmic domain. Parallel binding of FcγRIIb and the immunoreceptor tyrosine-based activation motif (ITAM) containing B cell receptor forms a negative feedback mechanism controlling antibody synthesis (13). Hyperactivated immune responses in FcγRIIb deficient mice, a lupus mouse model with an inhibitory-signaling defect, can become exhausted with sequential lipopolysaccharide (LPS) stimulation and thus possibly associated with a more severe sepsis, due partly to macrophage dysfunction (14). In their article, Jaroonwitchawan et al. have identified disruption of lipid metabolism in macrophages as a factor for severe endotoxin tolerance in the FcγRIIb-deficient lupus mice. Qiu et al. in their review addressed the role of organ-deposited IgG in causing multi-organ and tissue damage, and the importance of targeting IgG/FcγR signaling pathway in lupus.

Sphingolipids constitute a family of lipids, including sphingosine, ceramide, sphingosine-1-phosphate and ceramide-1-phosphate, which modulate various cell biological processes like growth regulation, cell migration, adhesion, apoptosis, senescence and inflammatory responses (15). Harden and Hammad, have discussed the advances made in sphingolipidomics as a diagnostic/prognostic tool for SLE and its co-morbidities. Using an integrated multi-omics approach, Huang et al. have reemphasized the role of dysregulated lipid metabolism, especially sphingolipid metabolism in contributing to SLE disease activity.

Immunomodulatory effects of diet and nutrients in SLE were discussed earlier (16). Among the fat-soluble vitamins, contribution of vitamin A and D in modulating SLE is well-recognized. All-trans retinoic acid (ATRA) is a bioactive lipid derived from vitamin A, which by interacting to its cognate nuclear receptors like retinoic acid receptor (RAR) and retinoid X receptor (RXR), functions as a regulator of gene transcription affecting the cell growth, differentiation, and apoptosis. ATRA is used to treat various forms of cancer. Although water insolubility, toxicity and bioavailability properties precludes its wider use, attempts are being made to reduce its undesirable properties by incorporating into liposomal formulations or nanodisks (17). Abdelhamid et al. have observed ATRA to differentially modulate lupus-associated kidney inflammation depending on the time of administration. Earlier reports from that group demonstrated ATRA supplementation exacerbated pre-existing autoimmunity in lupus affecting non-renal tissues like skin, lungs and brain. Taken together, ATRA effect on lupus seems to be dependent on specific phase of the disease and requires more studies using clinical samples. A significant environmental risk factor for SLE is vitamin D deficiency because it has a prominent role as an immune modulator and implicated in lupus pathophysiology (18). Vitamin D acts by regulating the genes and epigenome, through the active metabolite 1,25(OH)_2_D_3_ (calcitriol) binding to its receptor expressed by many immune cells. Kraemer et al. explored how low vitamin D intake promoted lupus pathology in lupus prone NZB/W F1 mice.

Several essential trace elements like iron, copper, zinc and selenium are required for the differentiation, activation and functioning of the immune cells, including T cells. Interestingly, conventional and regulatory T cells differ in their metabolism and show differential response to same stimulus. Earlier studies have shown that iron can direct CD4^+^ T-cells into a proinflammatory phenotype but Treg cells are resistant to iron deprivation. Pristane (2,6,10,14-tetramethylpentadecane) is a C19 isoalkane used as an inflammation inducing agent causing lupus, arthritis, and myeloma development in several rodents and pristane-induced lupus (PIL) is used as a classical animal model of SLE. Gao et al. have used PIL to study how Iron insufficiency promoted expansion of Treg cells by reducing ROS production and improved the clinical disease.

In chronic SLE patients, atherosclerosis and subsequent tissue damage can also be observed. Xing et al. have built an atherosclerotic risk prediction model in SLE patients that could be useful for clinicians. Tumurkhuu et al. investigated neutrophil contribution to endoplasmic reticulum (ER) stress in lung epithelial cells in the pristane induced diffuse alveolar hemorrhage, a rare complication of SLE. They have analyzed how ER stress can drive the pathology of pulmonary hemorrhage and neutrophils contribution in this process.

Exosomes are nano-sized vesicles released by cells under physiological and pathological conditions. They contain nucleic acids, proteins, lipids and metabolites. MicroRNAs (miRNAs) are short, regulatory, non-coding RNAs that act as regulators of gene expression and used in the diagnosis and treatment of various diseases. Importantly, most miRNAs in serum were found to be deposited in exosomes (19). The miR-451a is a cell metabolism-related miRNA, which was reported to have diagnostic value in certain cancers. Interestingly, Tan et al. demonstrated a correlation between downregulated serum exosomal miR-451a expression and SLE disease activity, renal damage as well as lymphocyte communication. In identifying active renal disease in SLE patients, Soliman et al. found urine:serum fractional excretion ratios are better than the corresponding urinary biomarker proteins.

Thus, this article collection was intended to highlight the substantial contributions published in this field with an aim to motivate researchers to do further advanced research leading to new findings and drug development that could ultimately alleviate the sufferings of lupus patients.

## Author contributions

KSN has written the manuscript and, both KSN and KN are topic editors. All authors approved the submitted version.

## References

[1] Wang YF Zhang Y Lin Z Zhang H Wang TY Cao Y. Identification of 38 novel loci for systemic lupus erythematosus and genetic heterogeneity between ancestral groups. Nat Commun (2021) 12:772. doi: 10.1038/s41467-021-21049-y 33536424 PMC7858632

[2] Fang Q Li T Chen P Wu Y Wang T Mo L. Comparative analysis on abnormal methylome of differentially expressed genes and disease pathways in the immune cells of RA and SLE. Front Immunol (2021) 12:668007. doi: 10.3389/fimmu.2021.668007 34079550 PMC8165287

[3] Kim HJ Verbinnen B Tang X Lu L Cantor H. Inhibition of follicular T-helper cells by CD8(+) regulatory T cells is essential for self tolerance. Nature (2010) 467:328–32. doi: 10.1038/nature09370 PMC3395240 20844537

[4] Mahajan A Herrmann M Munoz LE. Clearance deficiency and cell death pathways: A model for the pathogenesis of SLE. Front Immunol (2016) 7:35. doi: 10.3389/fimmu.2016.00035 26904025 PMC4745266

[5] Brodie EJ Infantino S Low MSY Tarlinton DM. Lyn, Lupus, and (B) lymphocytes, a lesson on the critical balance of kinase signaling in immunity. Front Immunol (2018) 9:401. doi: 10.3389/fimmu.2018.00401 29545808 PMC5837976

[6] Khan SQ Khan I Gupta V. CD11b activity modulates pathogenesis of lupus nephritis. Front Med (Lausanne) (2018) 5:52. doi: 10.3389/fmed.2018.00052 29600248 PMC5862812

[7] Yang WC Collette Y Nunes JA Olive D. Tec kinases: a family with multiple roles in immunity. Immunity (2000) 12:373–82. doi: 10.1016/S1074-7613(00)80189-2 10795735

[8] Kil LP de Bruijn MJ van Nimwegen M Corneth OB van Hamburg JP Dingjan GM. Btk levels set the threshold for b-cell activation and negative selection of autoreactive b cells in mice. Blood (2012) 119:3744–56. doi: 10.1182/blood-2011-12-397919 22383797

[9] Sim TM Ong SJ Mak A Tay SH. Type I interferons in systemic lupus erythematosus: A journey from bench to bedside. Int J Mol Sci (2022) 23:2505–21. doi: 10.3390/ijms23052505 PMC8910773 35269647

[10] Nandakumar KS. Targeting IgG in arthritis: Disease pathways and therapeutic avenues. Int J Mol Sci (2018) 19:677–97. doi: 10.3390/ijms19030677 PMC5877538 29495570

[11] Tsao BP Cantor RM Kalunian KC Chen CJ Badsha H Singh R. Evidence for linkage of a candidate chromosome 1 region to human systemic lupus erythematosus. J Clin Invest (1997) 99:725–31. doi: 10.1172/JCI119217 PMC507856 9045876

[12] Radstake TR Petit E Pierlot C van de Putte LB Cornelis F Barrera P. Role of fcgamma receptors IIA, IIIA, and IIIB in susceptibility to rheumatoid arthritis. J Rheumatol (2003) 30:926–33. 12734884

[13] Verbeek JS Hirose S Nishimura H. The complex association of FcgammaRIIb with autoimmune susceptibility. Front Immunol (2019) 10:2061. doi: 10.3389/fimmu.2019.02061 31681256 PMC6803437

[14] Ondee T Surawut S Taratummarat S Hirankarn N Palaga T Pisitkun P. Fc gamma receptor IIB deficient mice: A lupus model with increased endotoxin tolerance-related sepsis susceptibility. Shock (2017) 47:743–52. doi: 10.1097/SHK.0000000000000796 27849678

[15] Hannun YA Obeid LM. Sphingolipids and their metabolism in physiology and disease. Nat Rev Mol Cell Biol (2018) 19:175–91. doi: 10.1038/nrm.2017.107 PMC5902181 29165427

[16] Islam MA Khandker SS Kotyla PJ Hassan R. Immunomodulatory effects of diet and nutrients in systemic lupus erythematosus (SLE): A systematic review. Front Immunol (2020) 11:1477. doi: 10.3389/fimmu.2020.01477 32793202 PMC7387408

[17] Redmond KA Nguyen TS Ryan RO. All-trans-retinoic acid nanodisks. Int J Pharm (2007) 339:246–50. doi: 10.1016/j.ijpharm.2007.02.033 PMC2045639 17412536

[18] Iruretagoyena M Hirigoyen D Naves R Burgos PI. Immune response modulation by vitamin d: Role in systemic lupus erythematosus. Front Immunol (2015) 6:513. doi: 10.3389/fimmu.2015.00513 26528285 PMC4600954

[19] Ge Q Zhou Y Lu J Bai Y Xie X Lu Z. miRNA in plasma exosome is stable under different storage conditions. Molecules (2014) 19:1568–75. doi: 10.3390/molecules19021568 PMC6271968 24473213

